# Governing digital health transformation in South Africa: are policies, standards, and legislation fit for purpose?

**DOI:** 10.1093/oodh/oqag033

**Published:** 2026-09-04

**Authors:** Mandisa Msomi, Ngoako Marutha

**Affiliations:** Department of Information Science, University of South Africa (UNISA), Muckleneuk Campus, Preller Street, Muckleneuk Ridge, Pretoria, Gauteng 0002, South Africa; Department of Information Science, University of South Africa (UNISA), Muckleneuk Campus, Preller Street, Muckleneuk Ridge, Pretoria, Gauteng 0002, South Africa

**Keywords:** digital health transformation, digital health governance, policy and regulatory framework, ICT governance, interoperability, health information governance, provincial health departments, South Africa

## Abstract

This study examines the legislative and regulatory governance mechanisms influencing digital health transformation within Provincial Departments of Health in South Africa. It aimed to evaluate the effectiveness of existing legislative, regulatory, and policy frameworks in supporting digital health implementation and to develop a Provincial Digital Health Governance and Implementation Model to strengthen governance across provincial health departments. A qualitative multiple case study design, underpinned by the interpretivist paradigm, was employed. Data were collected through semi-structured interviews with Chief Information Officers and Information and Communication Technology (ICT) Managers from six Provincial Departments of Health and complemented by content analysis of key legislative, regulatory, and policy documents. Data were analysed thematically. The findings indicate that South Africa has established comprehensive legislative, regulatory, and policy frameworks to support digital health transformation; however, implementation remains inconsistent across provinces. Although these frameworks provide a foundation for digital health governance, their effectiveness is constrained by weak implementation mechanisms, limited institutional capacity, inconsistent ICT governance, inadequate infrastructure, insufficient digital skills, fragmented interoperability, and uneven compliance with information governance and information security standards. The study demonstrates that the principal challenge is not the absence of legislative and policy frameworks, but the governance capacity required for effective implementation. In response, the study proposes a Provincial Digital Health Governance and Implementation Model that integrates legislative and regulatory frameworks, health standards, institutional policies, governance structures, and implementation mechanisms to strengthen coordination, accountability, interoperability, regulatory compliance, and institutional readiness. The study contributes to digital health governance literature by providing a contextually relevant governance framework to support sustainable digital health transformation within South Africa’s provincial healthcare system.

## Introduction and background

The lack of service integration resulting from fragmented health information systems and weak policy coordination has contributed to increased verticalization within the healthcare sector. This fragmentation often encourages silo-based approaches and the independent implementation of policies and procedures, which ultimately results in inefficiencies and poor system performance (Malakoane et al. 2020). In this context, the establishment and effective implementation of a robust policy and regulatory framework for digital health transformation are essential for supporting coordinated planning, governance, and management of digital health initiatives that ultimately benefit society (OECD 2022, World Health Organization 2021). This study identifies a critical gap between the rapid pace of digital health transformation and the comparatively slow development of regulatory frameworks required to govern such innovations. This imbalance highlights the urgent need for stronger alignment between technological advancement and regulatory structures to ensure integrated and sustainable digital health implementation (Cachalia and Klaaren 2022). Currently, the pace of digital health transformation is outstripping the bureaucratic processes that guide policy formulation and regulatory oversight in the health sector (Cachalia and Klaaren 2022). This regulatory lag creates governance challenges that can hinder the effective implementation of digital health systems ( World Health Organization 2021, Cachalia and Klaaren 2022).

Legal and regulatory frameworks are critical for effective healthcare governance, particularly in environments where technological innovation advances more rapidly than legislative development (Cachalia and Klaaren 2021). In the healthcare sector, technological development frequently progresses faster than the evolution of legal frameworks designed to regulate it (Cachalia and Klaaren 2021). The rapid expansion of digital health technologies, including electronic health records, telemedicine, artificial intelligence, and mobile health platforms, creates significant legal, regulatory, and policy challenges (World Health Organization 2021). Despite this rapid transformation, legal systems are often characterized by slow legislative processes that struggle to keep pace with technological innovation (Cachalia and Klaaren 2021). However, legal frameworks remain dynamic and must continuously evolve to respond to disruptive technological developments by revisiting existing regulatory approaches and developing new governance mechanisms (Cachalia and Klaaren 2021, World Health Organization 2021).

Regulatory reform within the digital health sector plays an important role in shaping decision-making processes and supporting sustainable healthcare transformation (Fernandes and Chaltikyan 2020). Managing the transformation associated with digital health systems has become one of the most critical factors influencing the shift towards value-based healthcare and the broader functioning of healthcare systems (Fernandes and Chaltikyan 2020). Regulations can create enabling environments that support the effective delivery of healthcare services while ensuring patient safety and system accountability (Kruk et al. 2018). However, strong legal and regulatory frameworks must also encourage responsible innovation and facilitate the appropriate use of digital technologies in healthcare environments (Fernandes and Chaltikyan 2020).

Policy and regulatory frameworks therefore play a central role in guiding the implementation of digital health transformation (OECD 2022, World Health Organization 2021). At the same time, these frameworks must ensure the protection of individuals, sensitive health data, and critical digital infrastructure involved in digital health ecosystems (Bhavnani et al. 2017). Effective governance mechanisms are necessary to balance innovation with accountability, ensuring that digital health technologies are implemented ethically, securely, and in ways that strengthen healthcare systems (OECD 2022, World Health Organization 2021).

Despite the growing importance of policy and regulatory frameworks, existing literature has largely focused on describing legislation and regulatory instruments, with comparatively less attention given to how these frameworks are implemented and governed within healthcare institutions (OECD 2022, Cachalia and Klaaren 2022). The study therefore examines the policy and regulatory framework guiding digital health transformation within the healthcare sector, with particular attention to how governance mechanisms support or constrain the implementation of digitally enabled healthcare systems.

## Problem statement

South Africa has made considerable efforts to strengthen its healthcare system through the introduction of policies, legislation, and national strategies aimed at supporting digital health transformation. These initiatives seek to promote the use of digital technologies to improve healthcare delivery, enhance data management, and support integrated health services across the public healthcare sector (NDoH 2019, World Health Organization 2024). Despite these efforts, the governance and implementation of digital health systems across Provincial Departments of Health remain uneven and fragmented (Makeleni and Cilliers 2021, Zharima et al. 2023). Many digital health information systems continue to operate as stand-alone platforms with limited interoperability, thereby restricting effective data sharing and slowing progress towards the establishment of a unified national electronic health record (EHR) system (Makeleni and Cilliers 2021, Zharima et al. 2023).

Although legislative and regulatory frameworks, including the National Health Act and the National Digital Health Strategy, provide direction for coordinated health information systems and interoperability standards (NDoH 2019), evidence suggests that their implementation remains inconsistent across provinces (Zharima et al. 2023). Studies attribute these implementation challenges to variations in provincial governance capacity, infrastructure limitations, procurement processes, and shortages of technically skilled personnel required to implement, manage, and sustain digital health systems effectively (NDoH 2019, Zharima et al. 2023). Furthermore, the rapid advancement of digital technologies has outpaced the development of regulatory and governance mechanisms required to oversee digital health transformation, resulting in gaps in regulatory oversight, accountability, standards implementation, and compliance (World Health Organization 2021, Cachalia and Klaaren 2022, OECD 2022).

The lack of coherent alignment between legislative frameworks, health standards, and operational policies has further contributed to siloed information systems, weak interoperability between digital platforms, and inconsistent implementation of digital health initiatives across Provincial Departments of Health (Willie and Nkomo 2019, Zharima et al. 2023). These governance weaknesses also increase operational risks associated with fragmented digital health infrastructures (World Health Organization 2021, OECD 2022). For example, the 2024 ransomware cyberattack on the National Health Laboratory Service disrupted laboratory information systems and delayed the transmission of diagnostic results, demonstrating how weaknesses in governance, cybersecurity oversight, and digital resilience can affect the continuity of healthcare services (South African Government News Agency 2024, NHLS 2025).

Despite the existence of policies, legislative frameworks, health standards, and national digital health strategies, there remains limited empirical evidence regarding how these governance mechanisms collectively influence the implementation, coordination, regulatory oversight, compliance, and accountability of digital health transformation within Provincial Departments of Health in South Africa (NDoH 2019, World Health Organization 2021, OECD 2022). Consequently, there is insufficient understanding of how legislative frameworks, regulatory instruments, health standards, and operational policies are operationalized to support integrated, interoperable, and sustainable digital health systems within provincial healthcare settings. Addressing this knowledge gap is essential for strengthening digital health governance, improving compliance with health information standards, and supporting the effective implementation of integrated digital health systems across the public healthcare sector (World Health Organization 2021, OECD 2022).

## Purpose of the study

The purpose of this study was to examine the legislative and regulatory governance mechanisms guiding digital health transformation within Provincial Departments of Health in South Africa and to develop a Provincial Digital Health Governance and Implementation Model to strengthen governance and improve the implementation of digital health initiatives.

## Research objectives

- To assess the legislative and regulatory frameworks governing digital health transformation within Provincial Departments of Health in South Africa.
- To examine the health standards and compliance mechanisms supporting digital health transformation within Provincial Departments of Health in South Africa.
- To analyse the policies and procedures guiding the implementation of digital health transformation within Provincial Departments of Health in South Africa.
- To develop a provincial digital health governance and implementation model to strengthen digital health governance and implementation within Provincial Departments of Health in South Africa.

## Literature review

This section critically reviews the literature on digital health transformation, with particular emphasis on the legislative and regulatory frameworks, health standards and compliance, and policies and procedures that govern its implementation. Rather than merely describing existing legislation and policies, the review examines how this governance mechanisms influence the implementation, coordination, accountability, and sustainability of digital health initiatives. It further identifies gaps in the existing literature that the current study seeks to address.

## Legislative and regulatory frameworks for digital health transformation

The advancement of digital technologies has created significant opportunities to improve healthcare systems and strengthen service delivery globally (Fernandes and Chaltikyan 2020). However, the successful implementation of digital health transformation depends not only on technological innovation but also on legislative and regulatory governance mechanisms that facilitate implementation, accountability, and institutional coordination. The World Health Organization (WHO 2021) argues that digital health technologies require legislation and regulations that not only permit their use but also support their responsible, secure, and effective implementation. Similarly, the National Digital Health Strategy for South Africa (2019–24) emphasizes that legislative and regulatory frameworks provide the foundation for assessing the regulatory environment, strengthening governance arrangements, introducing new legislation where necessary, and promoting compliance. These frameworks therefore serve not only as legal instruments but also as governance mechanisms that shape digital health implementation.

Despite the recognized importance of legislative and regulatory governance, studies indicate that the legal environment supporting digital health remains underdeveloped in many countries. Al Meslamani (2023) notes that developers of digital health solutions frequently struggle to identify applicable regulations and understand how they should be operationalized. Similarly, Carnicero and Serra (2020) argue that the increasing integration of Information and Communication Technologies (ICTs) into healthcare requires governance models that combine political, administrative, and technological oversight. Their findings suggest that effective digital health transformation depends not only on legislative reform but also on governance systems capable of coordinating implementation and responding to rapidly evolving technologies.

The Global Strategy on Digital Health (2020–25) further emphasizes that legislation, regulations, and governance structures are fundamental to ensuring the sustainability, quality, safety, and security of digital health systems. Likewise, WHO (2021) recognizes health information as highly sensitive personal data requiring robust legal and governance safeguards. However, Erku et al. (2023) and Benis et al. (2021) observe that relatively few countries have established comprehensive legislative, regulatory, and governance frameworks capable of supporting digital health transformation. Cory and Stevens (2020) therefore argue that countries should adapt technological innovation to their own legal, institutional, and healthcare contexts to ensure effective governance and implementation.

Evidence from developed countries illustrates both progress and continuing governance challenges. Germany introduced the Digital Health Care Act of 2019 to accelerate healthcare digitalization (Glaser et al. 2020). Nevertheless, fragmented governance arrangements, dependence on paper-based systems, limited interoperability, and resistance from healthcare professionals continue to impede implementation (Nohl-Deryk et al. 2018, Caumanns 2019, Maass 2023, Schmitt 2023). Fernandes and Chaltikyan (2020) similarly conclude that digital health legislation in Germany and the broader European Union remains insufficiently comprehensive to support evolving digital health needs. Comparable governance challenges have been reported in Canada, where complex legislation limits the implementation of digital health innovations (Solomun et al. 2021). In the UK, although the UK General Data Protection Regulation (UK GDPR) strengthens the governance of personal health information (Kuan and Fulbright 2022), Guinchard (2021) found that compliance remains inconsistent because of weak implementation and enforcement mechanisms. Likewise, Ohannessian et al. (2020) argue that telemedicine regulation has not kept pace with rapid technological developments, illustrating the difficulty of maintaining responsive governance frameworks.

The literature reveals even greater governance challenges across Africa. Mburu and Kamau (2018) found that >67% of African countries lack dedicated digital health policies, limiting governance capacity and allowing digital health initiatives to be implemented with minimal regulatory oversight. Similarly, Alsamara and Farouk (2024) and Alunyu et al. (2020) argue that weak legislative and regulatory governance has contributed to fragmented digital health implementation across the continent. Compared with many African countries, South Africa has developed a relatively comprehensive legislative framework to support digital health transformation. The National Digital Health Strategy for South Africa (2019–24) recognizes that existing legislation provides a foundation that can be strengthened to support digital transformation. Key legislative instruments include the Constitution of the Republic of South Africa, the National Health Act, the Electronic Communications Security Act, the Electronic Communications and Transactions Act, the Electronic Communications Act, and the Protection of Personal Information Act (POPIA). Collectively, these legislative instruments regulate health service delivery, electronic communication, information governance, privacy, and data protection, thereby providing an important governance foundation for digital health transformation.

The Electronic Communications and Transactions Act support the use of digital technologies in healthcare by promoting innovation, confidentiality, privacy, and secure electronic transactions. The Electronic Communications Security Act strengthens the protection of critical digital infrastructure, while the Electronic Communications Act provides the legislative basis for connectivity and communication services required for digital health implementation. In addition, the National Archives and Records Service of South Africa Act establish principles for the management, security, and preservation of electronic health records (Marutha and Ngoepe 2018). Thulare et al. (2020) found that healthcare institutions frequently experience difficulties in producing reliable and trustworthy information, while Marutha and Ngoepe (2018) argue that noncompliance with records management legislation weakens the governance of electronic health records. Similarly, POPIA plays a central role in governing the privacy and confidentiality of personal health information. Staunton et al. (2020, 2021) demonstrate that POPIA significantly influences the governance of health information within digital health environments, whereas Buys (2017) and Adams et al. (2021) caution that poor compliance exposes healthcare institutions to legal, reputational, and ethical risks. Jones et al. (2019) further argue that although regulation is essential for patient safety, legislative frameworks should evolve alongside technological innovation to remain effective. Willie and Nkomo (2019) therefore conclude that responsive and adaptive regulatory governance is essential for South Africa’s digital transformation agenda.

Although the literature demonstrates that South Africa has established a comprehensive legislative and regulatory foundation for digital health transformation (National Digital Health Strategy for South Africa, 2019–2024, Willie and Nkomo 2019), relatively little empirical evidence exists on how these frameworks are interpreted, implemented, and operationalized within Provincial Departments of Health. Existing studies predominantly examine digital health legislation and policy from national, legal, or conceptual perspectives, focusing on the existence of legislative frameworks rather than their implementation in practice (Staunton et al. 2020, 2021, Jones et al. 2019). Moreover, the literature highlights persistent governance challenges, including weak compliance with records management legislation (Marutha and Ngoepe 2018), difficulties in maintaining reliable health information (Thulare et al. 2020), and the need for responsive regulatory frameworks to support digital transformation (Willie and Nkomo 2019). However, there is limited empirical evidence on the governance processes, institutional capacity, implementation practices, and coordination mechanisms that shape the operationalization of these frameworks within Provincial Departments of Health. Consequently, a significant knowledge gap remains regarding how legislative and regulatory governance mechanisms influence the implementation of digital health initiatives at the provincial level. This study addresses that gap by examining how legislative and regulatory governance mechanisms is interpreted, implemented, and operationalized within Provincial Departments of Health in South Africa.

## Health standards and compliance in digital health transformation

Health standards are widely recognized as critical governance mechanisms that support digital health transformation by promoting interoperability, accountability, quality assurance, and coordinated healthcare delivery (United Nations Industrial Development Organization 2021, United Nations Development Programme Astana Civil Service Hub 2023). Rather than serving merely as technical specifications, digital health standards establish common governance principles that enable healthcare organizations to exchange information consistently, protect patient data, and maintain system integrity across multiple institutions. The National Digital Health Strategy for South Africa (2019–24) defines digital health standards as approved specifications and protocols that guide the design, development, implementation, and operation of digital health systems. However, the effectiveness of these standards depends not only on their existence but also on institutional compliance, governance capacity, and continuous monitoring.

Interoperability standards have become central to digital health governance because they address fragmentation within health information systems while supporting integrated service delivery. Seneviratne (2023) argues that interoperability standards are essential for overcoming disconnected digital health systems and improving healthcare quality and value. Chatterjee et al. (2023) further note that these standards facilitate access to health information while protecting privacy and confidentiality. Nygren et al. (2023) found that countries aligning their standards with WHO guidance are better positioned to establish interoperable health systems and minimize duplication of digital health initiatives. These findings suggest that standards contribute to effective governance only when they are consistently adopted, implemented, and monitored across healthcare institutions.

Evidence from Africa indicates that governance challenges surrounding standards remain a significant barrier to digital health transformation. Tsegaye and Flowerday (2021) found that the absence of health information exchange policies and standards continues to hinder the implementation of eHealth across the continent. Similarly, Adebesin et al. (2013) argue that limited expertise in eHealth standardization constrains the ability of many African countries to adapt internationally recognized standards to local contexts. Mamuye et al. (2022) also identify poor interoperability, weak policy guidance, and limited evidence on existing health information exchange standards as persistent governance challenges. These weaknesses are compounded by inadequate digital literacy, shortages of skilled personnel, limited training, weak institutional coordination, and inequalities in healthcare infrastructure, all of which reduce organizational capacity to comply with established standards (Mamuye et al. 2022).

Poor governance of standards has broader implications for healthcare delivery. Ohia et al. (2023) and Olu et al. (2023) found that underutilization of digital health technologies at the primary healthcare level undermines service delivery and limits progress towards Universal Health Coverage. Likewise, Owusu and Gregar (2023) argue that fragmented health information systems contribute to inefficient resource utilization and unnecessary expenditure. Lokmic-Tomkins et al. (2023) further contend that limited evidence regarding large-scale implementation restricts informed governance decisions on digital health investment. Collectively, these studies suggest that technical standards alone cannot deliver digital transformation unless supported by effective governance, institutional commitment, and compliance mechanisms.

Internationally, standards such as Health Level Seven (HL7), OpenEHR, and ISO 13606 have become widely adopted because they provide governance frameworks for achieving interoperability rather than simply technical solutions. HL7 establishes internationally accepted standards for exchanging healthcare information between different systems (Noumeir 2019). Abril-Gonzalez et al. (2017) explain that HL7 enables interoperability by providing common standards rather than software applications. Similarly, the HL7 Reference Information Model supports structured communication and information exchange between healthcare systems (Smith and Ceusters 2006), while OpenEHR and ISO 13606 provide governance models for the consistent transfer, storage, and management of electronic health records (Weiss and Karamlou 2020). Pedrera-Jiménez et al. (2023) found that these standards improve interoperability by ensuring that healthcare information can be transferred, updated, and interpreted consistently across systems. Bourquard and Berler (2021) argue that semantic interoperability is particularly important because it ensures that exchanged information retains the same meaning across different healthcare platforms, thereby supporting continuity of care, clinical decision-making, and health data analytics.

In South Africa, the Health Normative Standards Framework (HNSF) provides the principal governance framework for interoperability within the public health sector. Wolmarans et al. (2014) explain that the HNSF establishes a shared enterprise architecture that enables electronic exchange of health information across healthcare facilities. The framework further supports the Health Information Exchange layer, facilitating standardized communication between applications, repositories, and health information registers. The revised Health Normative Standards Framework for Digital Health (2021) strengthens this governance approach by aligning interoperability standards with the National Digital Health Strategy for South Africa (2019–24). Consequently, the HNSF provides not only technical guidance but also governance mechanisms that promote consistency, accountability, and coordinated implementation of digital health systems across the public healthcare sector.

Despite the availability of internationally recognized standards such as HL7, OpenEHR, ISO 13606, and South Africa’s HNSF, the literature indicates that implementation and compliance remain inconsistent across healthcare systems (National Department of Health 2019, WHO 2021, OECD 2022). Existing studies have largely focused on describing interoperability standards, technical architectures, and information exchange models (Adebesin et al. 2013, WHO 2021), with comparatively limited attention given to the governance mechanisms required to coordinate implementation, monitor institutional compliance, and enforce adherence to these standards. Although studies have identified challenges relating to interoperability, fragmented information systems, and inconsistent implementation within South Africa’s public health sector (Katurura and Cilliers 2018, Zharima et al. 2023), there remains limited empirical evidence on how Provincial Departments of Health govern the implementation of interoperability standards, oversee compliance, and address implementation challenges in practice. This study addresses this gap by examining the governance of health standards and compliance within digital health transformation across Provincial Departments of Health in South Africa.

## Policies and procedures guiding digital health transformation

Policies and procedures are widely recognized as fundamental governance mechanisms for digital health transformation because they provide strategic direction, establish institutional responsibilities, and facilitate accountability during implementation (Dunn 2017, Foster and Bowie 2020). While policies define organizational goals and priorities, procedures operationalize these goals by providing structured processes that guide routine activities and support compliance with organizational and regulatory requirements (Burton and Obel 2018, McMeekin et al. 2020). Consequently, effective digital health transformation depends not only on the existence of policies but also on governance arrangements that ensure policies are translated into consistent institutional practices.

The Organisation for Economic Co-operation and Development (OECD 2020) argues that digital transformation presents complex governance challenges because of its broad social, economic, and institutional implications. Within the health sector, the WHO Global Strategy on Digital Health (2020–25) similarly emphasizes that governments should establish enabling policy environments capable of supporting coordinated, secure, and sustainable digital health systems. World Health Organization (n.d.) found that countries including South Africa, the UK, Germany, India, Kenya, Ethiopia, and the USA have developed digital health policies to address governance issues relating to data security, patient privacy, interoperability, infrastructure, reimbursement models, and public–private partnerships. Likewise, Stoumpos et al. (2023) argue that effective policy frameworks promote innovation, investment, health equity, and patient-centred care. However, these studies collectively suggest that policy effectiveness depends on governance mechanisms that support implementation, monitoring, and institutional accountability rather than policy formulation alone.

Despite growing policy development internationally, translating policy into practice remains a significant governance challenge. Abernethy et al. (2022) argue that developing policies for rapidly evolving digital technologies is inherently complex because technological innovation frequently outpaces policy development. Consequently, healthcare organizations often experience implementation gaps where policy objectives are not consistently translated into operational procedures and institutional practice (Abernethy et al. 2022). Similarly, Mburu and Kamau (2018) found that although many African countries have introduced digital health interventions to address health system challenges such as increasing disease burdens, workforce shortages, and service delivery constraints, these initiatives frequently operate without coherent policy guidance governing implementation, coordination, and accountability. This has contributed to fragmented digital health initiatives and inconsistent institutional practices across the continent (Mburu and Kamau 2018).

Evidence from African countries further demonstrates that governance capacity is essential for effective policy implementation. In Kenya, policymakers have developed digital health policy documents to respond to the rapid expansion of information and communication technologies within both the public and private sectors (Njoroge et al. 2017). However, policy effectiveness depends on institutional coordination and governance mechanisms capable of translating strategic intentions into operational practice (Njoroge et al. 2017). Similarly, Tadele Taye et al. (2022) found that successful digital health transformation in Ethiopia requires alignment between digital health initiatives, national health policies, sustainable financing, and collaboration among government stakeholders. These findings indicate that coherent governance structures, rather than policy development alone, determine whether digital health initiatives achieve their intended outcomes (Tadele Taye et al. 2022).

In South Africa, digital health transformation is supported by a national policy environment aimed at strengthening healthcare delivery through coordinated digital initiatives. Cachalia and Klaaren (2021) argue that the COVID-19 pandemic exposed weaknesses in existing legal and policy frameworks and highlighted the need for governance arrangements that are sufficiently adaptive to support digital technologies during public health emergencies. Similarly, the National Digital Health Strategy for South Africa (2019–24) identifies policy as a key governance mechanism for promoting health information exchange, interoperability, and coordinated digital transformation across the healthcare sector. However, although the strategy provides a clear national policy direction, its successful implementation depends on the extent to which Provincial Departments of Health translate national policy objectives into operational procedures, implementation plans, and institutional practices (National Digital Health Strategy for South Africa 2019–24).

Existing literature has predominantly focused on national policy frameworks and strategic intentions, with comparatively less attention given to how healthcare institutions develop operational procedures, governance mechanisms, and monitoring systems that facilitate policy implementation (Mburu and Kamau 2018, Abernethy et al. 2022). Furthermore, limited empirical evidence exists regarding how Provincial Departments of Health operationalize national digital health policies, monitor institutional compliance, coordinate implementation, and ensure accountability during digital health transformation (National Digital Health Strategy for South Africa 2019–24). This gap highlights the limited understanding of how policies and procedures governing digital health transformation are implemented within Provincial Departments of Health in South Africa. This study addresses this gap by examining their implementation in practice.

## Research methodology

This section outlines the research methodology adopted to examine the governance of digital health transformation within Provincial Departments of Health in South Africa. The methodology was guided by the study objectives, namely, to assess the legislative and regulatory frameworks, examine health standards and compliance mechanisms, analyse the policies and procedures governing digital health transformation, and develop a Provincial Digital Health Governance and Implementation Model. The study was grounded in the interpretivist research paradigm, underpinned by a constructivist ontological worldview. This paradigm was considered appropriate because it facilitated an exploration of how key stakeholders interpret and understand the legislative, regulatory, standards, and policy environment governing digital health transformation within provincial health departments. By examining participants’ experiences and perspectives, the study sought to develop an in-depth understanding of the governance processes, institutional practices, and implementation challenges influencing digital health transformation.

A qualitative research approach was employed because it enabled an in-depth exploration of governance structures, institutional processes, and implementation practices within Provincial Departments of Health. A multiple case study design was adopted, with Provincial Departments of Health serving as the cases. This design provided an opportunity to explore digital health governance across different provincial contexts and identify similarities and differences in the implementation of legislative, regulatory, standards, and policy frameworks. The study population comprised Chief Information Officers (CIOs) and ICT Managers, who are responsible for overseeing the governance and implementation of digital health initiatives within their respective departments. Purposive sampling was used to select participants with relevant knowledge and experience of digital health transformation. Of the nine Provincial Departments of Health, six provinces participated in the study, namely, KwaZulu-Natal, Mpumalanga, Free State, Northwest, Limpopo, and Northern Cape. Gauteng, Western Cape, and Eastern Cape did not participate because of nonresponse or the inability to confirm interview arrangements. Consequently, six participants, one representing each participating province, were interviewed.

Data were collected through semi-structured interviews and documentary content analysis. Semi-structured interviews were conducted with CIOs and ICT Managers to explore their perspectives on legislative and regulatory frameworks, health standards and compliance mechanisms, and policies and procedures governing digital health transformation. The interviews were conducted online via Microsoft Teams, audio-recorded with participants’ consent, and transcribed verbatim to preserve the accuracy of participants’ responses.

Documentary content analysis was undertaken to examine legislative, regulatory, standards, and policy documents relevant to digital health transformation in South Africa. The documentary sources were purposively selected based on their relevance to the study objectives and their contribution to understanding the governance of digital health transformation. An analytical framework derived from the research objectives guided the content analysis, enabling the systematic examination of governance principles, institutional responsibilities, implementation provisions, interoperability requirements, compliance mechanisms, policy implementation processes, and accountability arrangements. Documentary evidence was subsequently triangulated with interview data to compare governance provisions with implementation practices across Provincial Departments of Health. Table 1 summarizes the documentary sources analysed and the analytical focus applied during the content analysis.

**Table 1:** Documentary sources and analytical framework for qualitative content analysis.

Research objective	Documentary sources	Semi-structured interviews	Analytical focus
**1.** **Assess the legislative and regulatory frameworks governing digital health transformation within Provincial Departments of Health in South Africa.**	Constitution of the Republic of South Africa (1996); National Health Act (Act No. 61 of 2003); Protection of Personal Information Act (POPIA) (Act No. 4 of 2013); Promotion of Access to Information Act (PAIA) (Act No. 2 of 2000); Electronic Communications and Transactions Act (Act No. 25 of 2002); State Information Technology Agency (SITA) Act (Act No. 88 of 1998, as amended).	CIOs and ICT Managers provided perspectives on the implementation, interpretation, governance, and application of legislative and regulatory frameworks within Provincial Departments of Health.	Legislative mandates, governance principles, institutional responsibilities, implementation practices, information governance, and accountability mechanisms.
**2.** **Examine health standards and compliance governing digital health transformation within Provincial Departments of Health in South Africa.**	National Health Normative Standards Framework (HNSF); HL7; OpenEHR; ISO 13606.	CIOs and ICT Managers described the implementation of interoperability standards, compliance requirements, and challenges affecting health information exchange.	Standards governance, interoperability, compliance requirements, information exchange, data standardization, and implementation challenges.
**3.** **Analyse the policies and procedures governing digital health transformation within Provincial Departments of Health in South Africa.**	National Digital Health Strategy for South Africa (2019–24); Corporate Governance of ICT Policy Framework; Integrated ICT White Paper (2016); District Health Management Information System (DHMIS) Policy.	CIOs and ICT Managers explained policy implementation, institutional procedures, governance arrangements, coordination mechanisms, and operational practices.	Policy implementation, governance structures, institutional coordination, operational procedures, monitoring, oversight, and implementation practices.
**4.** **Develop a Provincial Digital Health Governance and Implementation Model.**	Synthesized evidence from all documentary sources analysed.	Synthesized evidence from all semi-structured interviews conducted with CIOs and ICT Managers.	Integration of documentary and interview evidence through thematic synthesis to identify recurring governance themes, implementation challenges, institutional coordination mechanisms, compliance practices, and accountability processes that informed the development of the Provincial Digital Health Governance and Implementation Model.

The data were analysed using thematic analysis, supported by ATLAS.ti software. Interview transcripts and documentary evidence were systematically coded using an initial coding framework derived from the study objectives and refined through iterative analysis. Recurring patterns were organized into themes corresponding to the legislative and regulatory frameworks, health standards and compliance, and policies and procedures governing digital health transformation. The integration of documentary and interview evidence facilitated methodological triangulation and strengthened the interpretation of governance processes and implementation practices across the participating Provincial Departments of Health.

Trustworthiness was enhanced through methodological triangulation, the maintenance of a comprehensive audit trail, and the application of the principles of credibility, dependability, confirmability, and transferability. These strategies enhanced the rigour and trustworthiness of the study.

The study acknowledged several limitations. Obtaining ethical approval and research permission from all nine Provincial Departments of Health proved challenging, resulting in the participation of only six provinces. Although this limited provincial representation, the participating provinces provided rich and diverse perspectives on digital health governance and implementation. Furthermore, scheduling interviews with CIOs and ICT Managers was constrained by their professional responsibilities, extending the data collection period to approximately nine months.

Ethical considerations were upheld throughout the study. Ethical approval was obtained from the University of South Africa Research Ethics Committee, and permission to conduct the study was granted by the participating Provincial Departments of Health. Participation was voluntary, and informed consent was obtained from all participants before data collection commenced. The researcher maintained the confidentiality, anonymity, and privacy of participants and ensured that the identities of both individuals and institutions were protected throughout the research process.

## Findings

This section presents the findings on the policy and regulatory framework governing digital health transformation within Provincial Departments of Health in South Africa. The analysis focused on the legislative, regulatory, standards, and policy instruments that guide the implementation and governance of digital health initiatives. The findings were generated through content analysis of relevant legislation, policies, and standards, complemented by interview data to provide a deeper understanding of how these frameworks are interpreted, implemented, and applied in practice within provincial health departments.

## Legislative and regulatory frameworks for digital health transformation

The findings revealed that participants regarded legislation and regulation as central to the implementation of digital health transformation. Participants identified several legal instruments as shaping the digital health environment in provincial health departments, with the most frequently cited being the State Information Technology Agency (SITA) Amendment Act 38 of 2002, the Protection of Personal Information Act (POPIA) 4 of 2013, the Promotion of Access to Information Act (PAIA) 2 of 2000, the National Health Act (NHA) 61 of 2003, and the Electronic Communications and Transactions (ECT) Act 25 of 2002. Collectively, these findings suggest that participants perceive legislative and regulatory frameworks not merely as legal requirements but as governance mechanisms that guide the implementation, coordination, and accountability of digital health transformation across provincial health departments.

The SITA Amendment Act emerged as the most prominent legislative framework in the interviews. Participants consistently described it as a critical instrument for enabling the technological environment necessary for digital health transformation. For example, one participant stated that *‘the State Information Technology Agency (SITA) Amendment Act 38 of 2002 is absolutely central to digital health transformation. It’s a framework that provides the technological foundation needed to align digital strategies with healthcare delivery’.* Similarly, another participant remarked that *‘the SITA Amendment Act forms the backbone of digital health legislation’.* These responses suggest that participants view SITA as an essential institutional vehicle for integrating ICT into healthcare delivery. However, despite this recognition, the findings also revealed concerns about the practical effectiveness of the SITA framework. Content analysis showed that while the Act establishes a specialised agency responsible for advancing information technology (IT) services across government departments, including healthcare, participants questioned the adequacy of its oversight and accountability. Interview data indicated that shortcomings in institutional performance and governance weaken the implementation of digital health initiatives. These findings indicate that the effectiveness of the SITA framework depends not only on its legislative mandate but also on the strength of institutional governance, oversight mechanisms, and accountability structures that support implementation across provincial health departments.

The POPIA was also identified as a major legislative instrument, particularly in relation to the privacy and confidentiality of patient information. Participants highlighted the importance of safeguarding personal health data in digital environments. One participant explained that *‘the Protection of Personal Information (POPI) Act 4 of 2013 is critical because it ensures that patient data remains secure and private in the digital age’.* While participants acknowledged its value in protecting personal information, the findings suggest that the Act does not fully address broader issues of digital health data governance, particularly around data ownership, control, and management. This indicates that although POPIA strengthens privacy protection, gaps remain in relation to the governance of digital health information across interoperable systems. The findings therefore highlight that effective digital health governance requires regulatory mechanisms that extend beyond privacy protection to encompass clear governance arrangements for data stewardship, accountability, and information sharing across integrated digital health platforms.

The PAIA was identified as another important legislative framework, particularly for transparency and access to health information. Participants noted that this legislation has relevance in digital health because it supports access to information held by public institutions. One participant stated that *‘the PAIA Act is key to promoting transparency and equitable access to health information, which is fundamental in a digital health ecosystem’.* Nonetheless, interview responses showed that its implementation is constrained by practical challenges, including limited public awareness, insufficient resources, and ambiguities around what constitutes public versus private information. Participants further described political interference as a significant obstacle. In this context, political interference referred to shifts in priorities due to political leadership changes, delays in implementation, and decisions influenced more by political interests than by evidence-based planning. These factors were perceived to undermine the consistency and sustainability of digital health reforms. These findings suggest that transparency legislation alone is insufficient to achieve effective digital health governance when implementation is constrained by institutional, political, and administrative factors that limit accountability and policy continuity.

The National Health Act was also widely recognized by participants as a cornerstone of healthcare governance in South Africa. Participants described it as providing the overall legal framework for the organization and administration of healthcare services. One participant explained that *‘the National Health Act 61 of 2003 is a cornerstone of healthcare regulation in South Africa. It provides the legal framework for healthcare governance, and it’s particularly relevant in digital transformation’.* Content analysis further showed that the Act supports the integration of digital health initiatives by clarifying the roles of national, provincial, and local government structures. However, participants indicated that implementation challenges remain, particularly in rural and underserved areas where administrative capacity and infrastructure are weaker. These findings demonstrate that while the Act establishes an enabling governance framework, disparities in institutional capacity and infrastructure continue to limit its consistent implementation across provincial health departments.

The ECT Act was similarly regarded as an important legal instrument for enabling digital health transformation. Participants associated it with regulating electronic communication and transactions within the healthcare sector. One participant observed that *‘the Electronic Communications and Transactions Act 25 of 2002 plays a vital role in ensuring the security and reliability of digital transactions in the health sector’.* The analysis confirmed that the Act provides legal clarity for electronic interactions and supports the expansion of digital health services. However, participants also noted limitations in its implementation, particularly regarding oversight and the uneven digital infrastructure across provinces. These findings suggest that legislative provisions supporting electronic transactions must be complemented by effective governance, institutional oversight, and equitable digital infrastructure to ensure consistent implementation across provincial health departments.

The findings demonstrate that while South Africa has a substantial legislative base for digital health transformation, the effectiveness of these legislative and regulatory frameworks depends largely on governance processes that support implementation, coordination, oversight, accountability, and institutional capacity. Weak oversight mechanisms, political interference, inadequate resources, and unresolved questions regarding digital health data governance continue to constrain the implementation of digital health transformation at the provincial level.

## Health standards and compliance in digital health transformation

The findings further revealed that health standards and compliance mechanisms are regarded as essential for guiding the implementation of digital health systems in provincial health departments. Participants consistently referred to the National Health Normative Standards Framework for Interoperability in e-Health (HNSF) and ISO standards, especially ISO/IEC 27001, as the key standards informing digital health transformation. Collectively, these findings suggest that participants perceive health standards not merely as technical requirements but as governance mechanisms that promote interoperability, accountability, and consistency in the implementation of digital health systems across provincial health departments.

Participants indicated that the HNSF provides the national framework for ensuring interoperability and reducing fragmentation across health information systems. One participant explained that *‘the Department of Health, in collaboration with the CSIR, has developed a comprehensive National Health Normative Standards Framework for Interoperability in e-Health. This framework serves as a guideline for developing digital health systems and addresses the fragmentation of existing systems within the country’.* This suggests that participants see the framework as critical for standardizing digital health system development across provinces. The findings further indicate that the HNSF performs an important governance function by providing a common framework that promotes coordination and interoperability across provincial health information systems, thereby reducing inconsistencies in system development and implementation.

The findings also highlighted the importance of communication and coordination between provinces during system development. Participants emphasized the need for compliance with nationally prescribed standards and recognized the role of the Council for Scientific and Industrial Research (CSIR) in monitoring adherence. One participant commented that *‘the inclusion of the CSIR in overseeing adherence to these standards during system construction is crucial to ensuring we stay on track’.* This indicates that external oversight and technical support are considered important in ensuring compliance with prescribed standards. These findings demonstrate that governance extends beyond the development of standards to include institutional oversight, monitoring, and coordination mechanisms that support consistent implementation across provinces.

In addition to national standards, participants referred to ISO/IEC 27001 as a significant international benchmark for information security. Participants regarded compliance with this standard as essential for safeguarding sensitive health data and ensuring secure digital health systems. One participant stated that *‘before implementing any new digital health system, it is imperative that we ensure it complies with both the National Health Normative Standards and the relevant ISO standards, particularly ISO/IEC 27001’.* These findings suggest that participants recognize information security as a critical component of digital health governance, with compliance serving to strengthen trust, accountability, and the protection of sensitive health information.

Despite the acknowledged importance of these standards, the findings show that compliance remains difficult in practice. Several participants reported challenges in fully implementing ISO/IEC 27001. For example, one participant noted that *‘our province follows the prescribed health criteria, but we do face challenges when it comes to complying with ISO/IEC 27001, particularly the guidelines related to information security’.* Another participant similarly observed that *‘we face challenges in fully complying with ISO/IEC 27001… due to internal resource constraints’.* These findings indicate that while provinces are aware of the standards and their importance, actual compliance is hampered by limited resources, technical complexity, and capacity constraints. This demonstrates that the existence of standards alone is insufficient to achieve effective digital health governance when institutional capacity and resource availability limit implementation.

Participants further indicated that governance, project management, and training structures have been put in place to support implementation. However, they also suggested that these measures remain insufficient without stronger institutional and financial support. These findings suggest that sustainable compliance depends not only on governance structures but also on adequate organizational capacity, skilled personnel, financial resources, and continuous institutional support. Overall, the findings demonstrate that while health standards are widely recognized as essential for promoting interoperability, information security, and consistency, their effective implementation remains uneven across provincial health departments due to persistent governance, resource, and capacity challenges.

## Policies and procedures guiding digital health transformation

The findings revealed that policies and procedures play an important role in translating legislative and standards requirements into operational practice within provincial health departments. Participants identified three key policy instruments guiding digital health transformation, namely, the Public Service Corporate Governance of ICT Policy Framework, the National Integrated ICT Policy White Paper, and the District Health Management Information System (DHMIS) Policy. Collectively, these findings suggest that participants perceive policies and procedures not merely as administrative instruments but as governance mechanisms that operationalize legislative and regulatory requirements and guide the implementation of digital health transformation across provincial health departments.

Participants described the Public Service Corporate Governance of ICT Policy Framework as essential for structuring ICT governance within provincial health departments. One participant stated that *‘the provincial health department strictly follows the structure outlined in the Public Service Corporate Governance of ICT Policy Framework’.* Another participant explained that the framework *‘plays a key role in shaping the ICT division’s structure and maintaining a unified approach to managing digital health projects’.* These responses suggest that the framework is seen as important for clarifying roles, responsibilities, and accountability in ICT governance. Content analysis showed that the framework is designed as a flexible governance instrument applicable across public administration. While this flexibility allows departments to adapt governance structures to their own contexts, it also means that implementation may vary considerably. This suggests that, although the framework provides broad guidance, the practical application of its principles depends heavily on departmental capacity and contextual adaptation. The findings therefore indicate that governance effectiveness depends not only on the existence of policy frameworks but also on the institutional capacity to implement governance principles consistently across provinces.

The National Integrated ICT Policy White Paper was also repeatedly identified as a key policy guiding digital health transformation. Participants emphasized its role in promoting system integration, interoperability, and alignment with national digital priorities. One participant explained that *‘the National Integrated ICT Policy serves as a cornerstone for our digital transformation initiatives. It emphasises the need for integrated systems’.* Another participant noted that *‘the policy mandates that any system we implement must be interoperable, which is essential in addressing the fragmentation of healthcare systems across provinces’.* These findings show that participants associate the White Paper with efforts to build interconnected systems and reduce duplication and inefficiency. They further suggest that the policy serves an important governance function by promoting coordinated digital health implementation and reducing fragmentation through standardized approaches to interoperability and system integration.

The DHMIS Policy emerged as another major policy framework supporting digital health transformation, particularly in the management of health information systems. Participants described it as essential for standardizing data management and improving information use in healthcare. One participant explained that *‘the DHMIS Policy plays a critical role in ensuring that health data is managed effectively and consistently’.* Another participant added that *‘the DHMIS Policy is fundamental to our work… it provides a standardised approach to managing health information’.* These findings indicate that participants view the DHMIS Policy as an important governance mechanism for promoting consistency, accountability, and standardization in health information management across provincial health departments.

Content analysis confirmed that the DHMIS Policy is aligned with the National Health Act and provides a regulatory framework for routine health information management. It outlines responsibilities at national, provincial, district, and facility levels and designates the Head of Department as responsible for oversight. It also provides for standard operating procedures to guide implementation. However, the analysis identified notable gaps in the policy. These include insufficient provisions for training and capacity building, limited monitoring and evaluation mechanisms, inadequate attention to data security and privacy, weak guidance on integration with other digital health systems such as telemedicine platforms and electronic health records, and a lack of clarity regarding resource allocation and sustainability. These findings suggest that although the policy establishes governance structures for health information management, weaknesses in implementation support and oversight mechanisms may limit its effectiveness in guiding digital health transformation.

A further finding was that participants did not always distinguish clearly between formal policy documents and local strategic plans. For instance, one participant remarked that *‘we have a provincial ICT strategic plan, and we are in the process of developing a custom digital health strategy… extracted from the National Digital Health Strategy’.* This suggests a lack of consistency in understanding the policy environment and points to uneven policy awareness across provinces. The findings indicate that variations in policy interpretation may contribute to inconsistent implementation and governance practices across provincial health departments.

Participants also described several mechanisms used to implement and reinforce policies and procedures. These included adapting national frameworks to provincial contexts, conducting environmental and risk assessments, assigning dedicated human resources, preparing compliance reports, using checklists, and relying on governance structures such as A Government Information Technology Officer (GITO). One participant explained that *‘the national framework serves as a guide, but we tailor our approach to the unique needs and environment of our province’.* Another stated that *‘implementing a policy effectively requires thorough documentation and monitoring… comprehensive reports on compliance… and checklists’.* These findings indicate that implementation is viewed as a contextual and managerial process rather than merely the adoption of policy documents. They further demonstrate that governance depends on institutional processes that support monitoring, accountability, risk management, and continuous compliance during implementation.

At the same time, some participants acknowledged that digital health policy implementation remains incomplete. One participant noted that *‘the digital health transformation process in our province is still in its early stages… we are still working on adapting and implementing (the frameworks) at the provincial level’.* Another participant stressed that *‘for digital health transformation to succeed… it’s essential to create an enabling environment’,* including dedicated personnel, sufficient resources, a clear implementation plan, and adequate ICT infrastructure. These findings demonstrate that the successful implementation of digital health policies depends not only on the availability of policy frameworks but also on governance conditions that support institutional readiness, resource mobilization, implementation capacity, and sustained organizational commitment. Overall, the findings suggest that while comprehensive policy instruments exist to guide digital health transformation, variations in institutional capacity, policy interpretation, governance practices, and implementation readiness continue to influence the consistency and effectiveness of digital health transformation across provincial health departments.

## Discussion

The findings demonstrate that although South Africa has established a comprehensive legislative and regulatory framework to support digital health transformation, the existence of legislation alone is insufficient to ensure effective implementation across provincial health departments. Rather, the findings suggest that the effectiveness of legislative frameworks depends on governance capacity, institutional readiness, accountability mechanisms, and the ability of provincial health departments to operationalize legislative requirements within diverse organizational contexts. This reinforces the view that digital health transformation is fundamentally a governance challenge rather than solely a legislative or technological undertaking. These findings support Fernandes and Chaltikyan (2020) and Jarrin and Parakh (2021), who argue that effective legal and regulatory frameworks provide the foundation for digital health innovation by promoting accountability, trust, and security. However, the present study extends these arguments by demonstrating that legislation achieves its intended outcomes only when supported by effective governance structures, institutional capacity, and implementation mechanisms.

The findings further demonstrate that rapid technological advancement has outpaced the capacity of existing governance and regulatory mechanisms to respond to emerging digital health challenges. Participants consistently highlighted implementation difficulties arising from inadequate institutional alignment, resource limitations, and inconsistent governance practices across provinces. These findings support Flannery and Jarrin (2018), who argue that regulatory frameworks must evolve continuously to remain responsive to technological innovation. The study therefore suggests that legislative frameworks should be viewed as dynamic governance instruments requiring continuous institutional adaptation, monitoring, and oversight to support sustainable digital health transformation.

## Digital health transformation legislation and regulations

The findings indicate that the SITA Amendment Act, POPIA, PAIA, the National Health Act, and the Electronic Communications and Transactions Act constitute the principal legislative frameworks governing digital health transformation within provincial health departments. Participants demonstrated a strong awareness of these legislative instruments, indicating that the legislative environment supporting digital health transformation is well established. However, the findings reveal that legislative awareness does not necessarily translate into effective implementation. This distinction highlights a critical governance issue: the effectiveness of legislation depends not only on its legal provisions but also on the institutional arrangements responsible for implementing, coordinating, and monitoring compliance across provincial health departments.

The SITA Amendment Act emerged as the most prominent legislative framework, with participants identifying it as the primary mechanism for enabling ICT infrastructure and supporting digital health implementation. While participants regarded the Act as providing the technological foundation necessary for digital transformation, they also raised concerns regarding institutional accountability, oversight, and organizational performance within SITA. These findings suggest that weaknesses within governance institutions responsible for implementing legislation may undermine the achievement of national digital health objectives. This finding supports Albertus and Hamman-Fisher (2021), who identified governance deficiencies within SITA, including organizational instability and reliance on external consultants. The present study extends this discussion by demonstrating that governance challenges within implementing institutions may reduce the effectiveness of otherwise well-developed legislative frameworks, thereby limiting the successful implementation of digital health initiatives across provincial health departments.

Participants also identified POPIA as a critical legislative instrument for protecting the privacy and confidentiality of patient information within digital health environments. While participants acknowledged the Act’s contribution to safeguarding personal information, they consistently indicated that it provides limited guidance on broader digital health governance issues, particularly concerning data ownership, stewardship, interoperability, and the management of health information across integrated digital platforms. These findings suggest that privacy legislation alone is insufficient to address the broader governance requirements of integrated digital health ecosystems. This finding corroborates Brand et al. (2023) and Katurura and Cilliers (2018), who argue that South Africa’s digital health data governance framework remains fragmented. The present study further demonstrates that strengthening digital health governance requires regulatory frameworks that balance privacy protection with clear governance arrangements for data sharing, accountability, stewardship, and interoperability.

Similarly, the findings regarding PAIA indicate that although the Act provides an important legislative basis for transparency and access to health information, its implementation is constrained by institutional and political factors. Participants identified limited resources, inadequate public awareness, implementation delays, and political interference as significant barriers to effective implementation. These findings suggest that transparency legislation cannot achieve its intended governance objectives without adequate institutional capacity and enforcement mechanisms. This supports Taylor (2019), who argues that weak enforcement processes reduce the practical effectiveness of information access legislation. The present study adds that governance effectiveness depends not only on legislative provisions but also on the institutional environment responsible for implementing those provisions consistently and transparently.

The National Health Act was similarly recognized as the overarching legislative framework governing healthcare delivery and digital health transformation in South Africa. Participants acknowledged its importance in defining governance responsibilities across national, provincial, and local government structures. However, they also highlighted persistent implementation challenges within rural and underserved provinces, where limited infrastructure and administrative capacity continue to constrain implementation. These findings suggest that decentralized implementation requires governance systems capable of responding to contextual differences in institutional capacity. This finding aligns with Haricharan et al. (2021) and Panya and Abuya (2023), who identify structural inequalities and governance constraints as barriers to equitable healthcare delivery. The present study further demonstrates that strengthening digital health governance requires greater attention to institutional readiness and implementation capacity across provincial health departments.

The findings further indicate that the Electronic Communications and Transactions Act provide the legal foundation for electronic communication and digital transactions within healthcare. Participants associated the Act with strengthening the legitimacy, security, and reliability of digital interactions. However, they also acknowledged that implementation remains uneven because governance mechanisms supporting oversight, monitoring, and digital infrastructure differ considerably across provinces. These findings suggest that legislative provisions supporting digital transactions are necessary but insufficient in the absence of institutional governance arrangements capable of ensuring consistent implementation. This supports Prahladh and Van Wyk (2022) and Vidal-Alaball et al. (2024), who emphasize the importance of electronic transaction legislation in enabling digital healthcare services. The present study extends this argument by demonstrating that legislative effectiveness depends on governance systems capable of supporting implementation, regulatory oversight, and institutional accountability.

The findings demonstrate that South Africa possesses a comprehensive legislative framework capable of supporting digital health transformation. However, the study reveals that governance challenges, including weak institutional oversight, inconsistent implementation, political interference, limited organizational capacity, resource constraints, and unresolved digital health data governance issues, continue to undermine the translation of legislative intent into effective practice. Consequently, the findings suggest that future digital health reforms should move beyond strengthening legislation alone and place greater emphasis on improving governance capacity, institutional accountability, implementation coordination, and regulatory oversight to achieve sustainable digital health transformation across provincial health departments.

## Health standards and compliance in digital health transformation

The findings demonstrate that health standards and compliance frameworks play a critical role in governing the implementation of digital health systems within provincial health departments. Participants consistently identified the HNSF as the principal mechanism for promoting interoperability, reducing fragmentation, and standardizing digital health system development across provinces. These findings suggest that health standards function not only as technical specifications but also as governance mechanisms that promote coordination, accountability, and consistency in digital health implementation. This finding supports Adebesin et al. (2013), who argue that interoperability standards are fundamental for integrating health information systems, and aligns with the National Department of Health (2019), which positions the HNSF as the national framework for achieving interoperable digital health systems. Similarly, the World Health Organization (2021, 2024) emphasizes that interoperability standards are essential for strengthening governance, improving continuity of care, and enabling integrated digital health ecosystems. The present study extends this body of knowledge by demonstrating that the successful implementation of interoperability standards depends not only on the existence of the HNSF but also on governance mechanisms that facilitate institutional coordination, oversight, and consistent implementation across provincial health departments.

Participants further highlighted the important role of the CSIR in supporting compliance with nationally prescribed standards during system development. This finding indicates that external oversight and technical support constitute important governance mechanisms for strengthening compliance and promoting accountability during digital health implementation. The findings therefore reinforce the argument by the OECD (2022) that effective governance requires institutions capable of monitoring compliance, coordinating implementation, and ensuring accountability across multiple stakeholders rather than relying solely on regulatory requirements.

Despite the existence of national standards, the findings reveal that compliance with ISO/IEC 27001 remains a significant challenge within provincial health departments. Participants consistently reported that limited financial resources, inadequate technical capacity, and insufficient institutional support constrain the implementation of internationally recognized information security standards. These findings indicate that awareness of standards alone is insufficient to achieve compliance where governance capacity and organizational readiness remain weak. Similar implementation challenges have been reported by Longras et al. (2018), who identify financial, organizational, and technical barriers to implementing ISO standards. Likewise, the World Health Organization (2021) recognizes that successful implementation of digital health standards requires sustained investment in institutional capacity, workforce development, and governance structures. The present study further demonstrates that compliance should be understood as a governance process requiring institutional commitment, effective leadership, continuous monitoring, and adequate resource allocation rather than merely adherence to technical requirements.

The findings also highlight the importance of information security governance in digital health transformation. Participants associated ISO/IEC 27001 with protecting sensitive health information yet acknowledged persistent implementation challenges that may expose provincial health information systems to cybersecurity risks. These findings reinforce Cachalia and Klaaren (2022), who argue that advances in digital technologies have outpaced the development of governance and regulatory mechanisms needed to safeguard digital health environments. They also support the OECD (2022), which emphasizes that cybersecurity resilience depends on governance systems that integrate risk management, accountability, and regulatory oversight into digital transformation initiatives. The present study therefore suggests that strengthening information security requires governance frameworks that integrate technical standards with effective institutional oversight, continuous risk assessment, and organizational accountability.

The findings demonstrate that while South Africa has established comprehensive national standards to guide digital health transformation, their effectiveness depends on governance systems capable of supporting implementation, compliance, institutional coordination, and continuous oversight. This finding corroborates Makeleni and Cilliers (2021) and Zharima et al. (2023), who identify fragmented health information systems and inconsistent implementation as persistent barriers to digital health transformation. However, the present study extends previous research by demonstrating that these implementation challenges are fundamentally governance issues arising from variations in institutional capacity, compliance monitoring, and organizational readiness across provincial health departments. Consequently, strengthening digital health transformation requires not only the development of robust standards but also governance mechanisms that enable provinces to implement, monitor, and sustain compliance consistently across the public healthcare sector.

## Policies and procedures in digital health transformation

The findings demonstrate that policy frameworks play a critical role in translating legislative and regulatory requirements into operational practice within provincial health departments. Participants consistently identified the Public Service Corporate Governance of ICT Policy Framework, the National Integrated ICT Policy White Paper, and the DHMIS Policy as the principal policy instruments guiding digital health transformation. These findings suggest that policies and procedures function not merely as administrative documents but as governance mechanisms that operationalize legislation, clarify institutional responsibilities, and support accountability during digital health implementation. This finding aligns with the National Department of Health (2019), which emphasizes that policy frameworks are essential for translating national digital health objectives into coordinated implementation across the health sector. Similarly, the World Health Organization (2021, 2024) argues that effective digital health transformation depends on governance arrangements that integrate legislation, standards, and operational policies to ensure consistent implementation. The present study extends this understanding by demonstrating that policy effectiveness is influenced by institutional capacity and governance practices at the provincial level.

Participants identified the Public Service Corporate Governance of ICT Policy Framework as the primary policy guiding ICT governance within provincial health departments. The findings indicate that the framework provides clarity regarding governance structures, institutional roles, responsibilities, and accountability mechanisms for managing digital health initiatives. However, participants also reported variations in how the framework is implemented across provinces. These findings suggest that governance effectiveness depends not only on the availability of policy frameworks but also on the institutional capacity to operationalize governance principles consistently. This finding supports Nxozi and Flowerday (2021), who identified weaknesses in ICT governance controls within South African public sector institutions. The findings further reinforce the OECD (2022), which argues that governance frameworks achieve their intended outcomes only when supported by effective oversight, institutional accountability, and implementation capacity.

The National Integrated ICT Policy White Paper emerged as an important strategic framework promoting interoperability, system integration, and alignment with national digital priorities. Participants regarded the policy as essential for reducing fragmentation and guiding the development of integrated digital health systems. Nevertheless, the findings reveal considerable variation in how provinces interpret and implement the policy, resulting in inconsistent governance practices. These findings suggest that policy coherence depends not only on strategic direction but also on governance mechanisms that promote coordinated implementation across institutions. This observation supports the National Department of Health (2019), which identifies interoperability as a national priority for digital health transformation, and reinforces the World Health Organization (2024), which highlights policy coordination as a prerequisite for integrated digital health systems. The present study contributes by demonstrating that differences in provincial implementation remain an important governance challenge despite the existence of national policy guidance.

The findings further demonstrate that the DHMIS Policy serves as a critical governance framework for health information management by promoting standardized data management and supporting evidence-based decision-making within provincial health departments. Participants recognized the policy’s contribution to improving the consistency and quality of health information. However, content analysis and interview findings identified several implementation challenges, including insufficient training, limited monitoring and evaluation mechanisms, inadequate guidance on interoperability and data security, and uncertainty regarding long-term sustainability. These findings suggest that effective policy implementation requires governance mechanisms that extend beyond policy development to include continuous institutional support, monitoring, and capacity development. This finding supports Kumalo (2006) and Dehnavieh et al. (2018), who argue that health information systems depend on strong governance, institutional capacity, and sustainable infrastructure. Similarly, the World Health Organization (2021) emphasizes that successful digital health implementation requires ongoing investment in governance, workforce development, and organizational capacity rather than relying solely on policy frameworks.

An important finding of this study is that participants did not always distinguish between formal policy frameworks and provincial strategic plans. This inconsistency suggests uneven policy awareness and understanding across provincial health departments, which may contribute to differences in implementation and governance practices. These findings indicate that governance challenges are influenced not only by policy design but also by the extent to which policy actors understand and operationalize policy requirements within their respective institutional contexts. Similar observations have been reported by Hovenga and Hovenga (2022), who found that healthcare professionals often recognize the importance of digital transformation while remaining unfamiliar with the governance frameworks that support its implementation. The present study extends this argument by demonstrating that policy awareness is itself a governance issue influencing implementation consistency across provincial health departments.

The findings demonstrate that South Africa has established comprehensive policy frameworks to support digital health transformation. However, the effectiveness of these frameworks depends on governance systems capable of ensuring institutional coordination, policy coherence, implementation capacity, accountability, monitoring, and sustained organizational support. Consistent with Cachalia and Klaaren (2022), OECD (2022), and the World Health Organization (2021, 2024), the study demonstrates that successful digital health transformation requires governance mechanisms that integrate legislation, standards, and operational policies into coherent implementation practices. The study therefore contributes to the growing digital health governance literature by showing that the principal challenge is not the absence of policy frameworks but the uneven governance capacity required to implement them consistently across provincial health departments.

## Conclusion

This study examined the policy and regulatory framework governing digital health transformation within provincial Departments of Health in South Africa. The findings demonstrate that although comprehensive legislative, regulatory, and policy instruments, including the SITA Amendment Act, POPIA, PAIA, the National Health Act, the National Digital Health Strategy for South Africa (2019–24), the HNSF, and supporting ICT governance policies provide a foundation for digital health transformation, their implementation across provincial health departments remains uneven. The study revealed that the existence of legislative and policy frameworks alone does not guarantee effective digital health transformation. Rather, their effectiveness depends on governance mechanisms that promote institutional coordination, accountability, regulatory oversight, compliance, and implementation capacity.

The findings further revealed that although provincial health departments have developed digital health strategies aligned with national legislation and policy frameworks, implementation remains constrained by limited institutional readiness and governance capacity. Weak compliance mechanisms, inconsistent policy implementation, and ambiguities in information governance, particularly regarding the distinction between public and private information under PAIA, continue to impede the operationalization of digital health initiatives. Furthermore, variations in ICT governance practices, limited integration between healthcare services and ICTs, and the absence of clearly defined implementation programmes contribute to the persistent gap between policy intentions and implementation outcomes. These governance challenges are further compounded by inadequate digital skills, infrastructure limitations, unreliable energy supply, and workforce capacity constraints, which continue to affect the consistent implementation of digital health systems across provinces.

The study also highlights the importance of health standards and compliance in supporting digital health transformation. Although participants recognized the value of the National Health Normative Standards Framework and ISO/IEC 27001 in promoting interoperability, information security, and standardization, compliance with these standards remains inconsistent because of financial, technical, and institutional resource limitations. These findings suggest that strengthening compliance requires more than the existence of standards; it requires governance systems capable of supporting institutional oversight, continuous monitoring, workforce development, and sustained organizational commitment.

Overall, the study demonstrates that the principal challenge facing digital health transformation in South Africa is not the absence of legislative, regulatory, and policy frameworks but the governance capacity required to implement them effectively across provincial health departments. The study therefore contributes to the growing body of digital health governance literature by demonstrating that effective digital health transformation depends on strengthening institutional governance, improving implementation coordination, enhancing regulatory oversight and accountability, and ensuring sustained investment in organizational capacity. Accordingly, future policy interventions should prioritize strengthening ICT governance, improving institutional readiness, enhancing digital skills and infrastructure, reinforcing information security and compliance mechanisms, and providing clearer operational guidance to support the sustainable implementation of integrated and interoperable digital health systems across South Africa’s provincial healthcare sector.

## Recommendations

This section presents recommendations derived from the analysis and interpretation of the study findings on the policy and regulatory framework governing digital health transformation within Provincial Departments of Health in South Africa. The recommendations are informed by the governance, implementation, compliance, and institutional challenges identified in the study and are intended to strengthen the effective implementation and long-term sustainability of digital health transformation. Central to these recommendations is the development of a Provincial Digital Health Governance and Implementation Model that is responsive to the operational realities, governance structures, and institutional capacities of provincial health departments. The proposed model should provide a coherent governance framework that aligns provincial digital health implementation with national legislation, regulatory requirements, health standards, and international best practices while remaining adaptable to diverse provincial contexts. Such a model would strengthen institutional governance, improve regulatory compliance, enhance accountability, and promote coordinated implementation of digital health systems across provincial healthcare institutions.

The proposed model should incorporate practical and measurable governance mechanisms that strengthen implementation while ensuring alignment with national legislation and internationally recognized digital health standards. The findings demonstrate that effective implementation depends on institutional readiness as much as legislative compliance. Accordingly, provincial health departments should strengthen the integration of human resources and ICTs by investing in digital leadership, multidisciplinary governance structures, and workforce development. Targeted education and continuous professional development programmes should be implemented to improve digital competencies among healthcare professionals, ICT practitioners, and managers responsible for digital health implementation. These initiatives should be supported by clear implementation plans, defined accountability structures, and continuous monitoring to ensure consistent application of digital health policies across provinces.

The study further recommends sustained investment in digital infrastructure to support the implementation of secure, integrated, and resilient digital health systems. Provincial health departments should prioritize reliable ICT infrastructure, secure digital platforms, stable energy supply, and adequate financial resources to strengthen organizational readiness for digital health transformation. In addition, existing legislative and policy frameworks should be periodically reviewed to provide clearer operational guidance on information governance, interoperability, cybersecurity, data stewardship, and the management of sensitive health information. Strengthening these governance arrangements would reduce implementation inconsistencies and improve institutional accountability across provincial health departments.

The establishment of standardized ICT governance mechanisms aligned with international best practices, including the HNSF and ISO/IEC 27001, is recommended to strengthen compliance, accountability, and institutional coordination. However, these governance mechanisms should be sufficiently flexible to accommodate the varying institutional capacities and contextual realities of individual provinces. Strengthening interoperability through open system architectures and standardized information governance practices would improve data sharing, reduce system fragmentation, enhance information security, and support integrated service delivery across the public healthcare sector. Aligning provincial implementation strategies with a coherent national digital health governance approach would further promote policy consistency, strengthen implementation coordination, and reduce disparities in digital health implementation across provinces.

Finally, the proposed Provincial Digital Health Governance and Implementation Model should integrate legislative and regulatory requirements, health standards, organizational governance, implementation processes, and emerging digital technologies within a single governance framework. The model should incorporate mechanisms for continuous monitoring, evaluation, compliance auditing, and stakeholder engagement to strengthen implementation accountability and support evidence-based decision-making. By strengthening governance capacity, regulatory oversight, institutional coordination, and organizational readiness, the proposed model has the potential to improve the ethical and secure management of health information, strengthen public trust, support integrated and interoperable digital health systems, and contribute to the sustainable digital transformation of South Africa’s provincial healthcare sector.

## Proposed provincial digital health governance and implementation model

The Proposed Provincial Digital Health Governance and Implementation Model was developed from the findings of this study to address the governance, implementation, and institutional challenges influencing digital health transformation within Provincial Departments of Health in South Africa. The findings demonstrated that although comprehensive legislative, regulatory, and policy frameworks exist, their implementation remains inconsistent because of variations in governance capacity, institutional readiness, regulatory compliance, and coordination across provinces. The model therefore provides a governance framework that integrates legislative and regulatory requirements, health standards, institutional policies, and governance mechanisms into a structured approach for implementing and sustaining digital health transformation. As illustrated in Table 2, the model seeks to strengthen governance by promoting accountability, institutional coordination, regulatory compliance, and implementation consistency across provincial health departments.

**Table 2:** Proposed provincial digital health governance and implementation model.

Components	Description	Key functions
**1.** **Policy and Regulatory Governance**	Provides the overall governance structure for digital health transformation in Provincial Departments of Health, ensuring alignment between legislation, policy, and implementation.	Alignment with national health legislation and policy; integration of the SITA Amendment Act, POPIA, PAIA, National Health Act, National Digital Health Strategy, and international standards such as ISO/IEC 27001; strengthening regulatory coherence and implementation governance.
**2.** **Unified National Digital Health System**	Establishes an integrated national digital health environment that connects provincial digital health initiatives through interoperable systems.	Promotes interoperability; enables integrated health information platforms; reduces fragmentation and duplication; supports coordinated service delivery and evidence-based decision-making.
**3.** **Digital Health Policies and Procedures**	Provides operational guidance for implementing, managing, and expanding digital health initiatives across provincial health departments.	Supports information governance, cybersecurity, telemedicine, eHealth technologies, patient rights, implementation planning, stakeholder engagement, and alignment with national and international best practices.
**4.** **Health Standards and Compliance**	Ensures compliance with national and international digital health, interoperability, and information security standards.	Supports interoperability; strengthens information security and data governance; protects sensitive health information; improves data quality, reliability, and secure information exchange.
**5.** **Clearly Defined Institutional Roles**	Establishes governance responsibilities for national, provincial, and district stakeholders involved in digital health transformation.	National Department of Health provides strategic leadership and policy direction; Provincial Departments implement and contextualize policies; district structures support operational implementation; promotes accountability and coordinated resource utilization.
**6.** **Oversight and Institutional Accountability**	Strengthens governance oversight through continuous monitoring, evaluation, compliance, and institutional accountability.	Performance monitoring and evaluation; compliance auditing; strengthened institutional oversight; transparent resource allocation; continuous improvement of digital health governance and implementation.

The first component of the model focuses on policy and regulatory governance. The findings revealed that legislation such as the SITA Amendment Act, POPIA, PAIA, the National Health Act, and supporting digital health policies provide an important regulatory foundation for digital health transformation; however, implementation remains uneven across provinces. The model therefore emphasizes the continuous review, alignment, and strengthening of legislative and regulatory frameworks to improve policy coherence, clarify governance responsibilities, and support effective implementation. By identifying regulatory gaps, overlaps, and implementation challenges, this component promotes stronger governance, improved institutional accountability, and greater consistency in implementing digital health initiatives.

The second component addresses health standards and compliance**,** which emerged as a critical governance requirement for achieving secure, interoperable, and integrated digital health systems. The findings demonstrated that compliance with the HNSF and international standards such as ISO/IEC 27001 remains constrained by limited financial resources, inadequate technical capacity, and inconsistent institutional support. Consequently, the model positions compliance as a governance process rather than merely a technical requirement. Strengthening compliance mechanisms will enhance interoperability, improve information security, support effective data governance, and promote the secure exchange of health information across provincial health departments. The model further incorporates institutional policies and procedures to address the policy implementation challenges identified in the study. Although participants recognized the importance of policies such as the Public Service Corporate Governance of ICT Policy Framework, the National Integrated ICT Policy White Paper, and the DHMIS Policy, the findings revealed inconsistencies in policy awareness, interpretation, and implementation across provinces. The model therefore recommends strengthening institutional governance through clearly defined operational policies, implementation guidelines, monitoring mechanisms, and accountability structures that facilitate consistent policy implementation while remaining responsive to provincial contexts.

Recognizing that fragmented digital health systems remain a significant barrier to effective service delivery, the model also promotes the establishment of a unified national digital health system supported by interoperable digital platforms and standardized information governance practices. Integrating provincial digital health systems within a coordinated national governance framework would reduce duplication, improve information sharing, strengthen continuity of care, and support evidence-based decision-making throughout the healthcare system. The model further emphasizes clearly defined institutional roles and responsibilities for digital health governance. The findings indicate that uncertainty regarding governance responsibilities contributes to inconsistent implementation across provincial health departments. Clearly distinguishing the strategic responsibilities of the National Department of Health from the operational responsibilities of Provincial Departments of Health and district-level structures would strengthen accountability, improve coordination, and promote more efficient utilization of institutional resources during digital health implementation.

Finally, the model strengthens oversight and institutional accountability, which emerged as one of the principal governance challenges identified in the study. The findings demonstrated that weak monitoring systems, inconsistent compliance practices, and limited governance oversight contribute to implementation gaps despite the existence of comprehensive legislative and policy frameworks. Accordingly, the model proposes continuous monitoring and evaluation, performance assessment, compliance auditing, and strengthened institutional accountability as core governance mechanisms for improving implementation effectiveness. Strengthening the oversight role of institutions responsible for digital health governance, including SITA and other relevant governance structures, would enhance transparency, improve regulatory compliance, and support the sustainable implementation of digital health initiatives across provincial health departments.

Collectively, the components of the Proposed Provincial Digital Health Governance and Implementation Model provide an integrated governance framework that responds directly to the challenges identified in this study. Rather than proposing additional legislation or policy instruments, the model demonstrates that strengthening digital health transformation depends on improving governance capacity, institutional coordination, implementation oversight, regulatory compliance, organizational readiness, and accountability. By integrating legislative and regulatory frameworks, health standards, institutional policies, governance structures, and implementation mechanisms into a unified governance framework, the proposed model provides a practical and contextually responsive approach to strengthening digital health transformation within South Africa’s Provincial Departments of Health. The components of the proposed Provincial Digital Health Governance and Implementation Model are presented in Table 2.

## Implication of the study

The findings of this study have important implications for digital health governance, policy development, and implementation within South Africa’s provincial health departments. The study demonstrates that the effectiveness of digital health transformation depends less on the existence of legislative and policy frameworks than on the governance capacity to implement them consistently. This highlights the need for a more integrated and standardized governance approach that strengthens institutional coordination, regulatory oversight, accountability, and implementation capacity to reduce disparities in digital health implementation across provinces. The findings further imply that strengthening institutional readiness is essential for sustainable digital health transformation. Effective implementation requires governance structures that support organizational capacity, leadership, workforce development, stakeholder engagement, and change management. In addition, stronger collaboration between government, healthcare institutions, academic institutions, and research organizations is necessary to promote innovation and support evidence-informed policy and practice.

The study also underscores the importance of strengthening governance oversight through effective monitoring and evaluation mechanisms. Clear performance indicators, compliance audits, and continuous monitoring would enhance accountability, improve resource utilization, and support the consistent implementation of digital health initiatives in line with national priorities. The findings suggest that strengthening governance arrangements, institutional capacity, and implementation mechanisms will enable policymakers and healthcare managers to address persistent systemic challenges affecting digital health transformation. These insights provide practical guidance for future governance reforms and policy implementation aimed at achieving sustainable, integrated, and interoperable digital health systems within South Africa’s provincial healthcare sector.

## Final conclusion

This study examined the policy and regulatory framework governing digital health transformation within Provincial Departments of Health in South Africa. The findings demonstrate that although South Africa has established comprehensive legislative, regulatory, and policy frameworks to support digital health transformation, their implementation remains inconsistent across provinces. The study revealed that the principal challenge is not the absence of these frameworks but the governance capacity required to implement them effectively. Weak implementation mechanisms, limited ICT governance compliance, inadequate infrastructure, insufficient digital skills, and uneven institutional capacity continue to constrain the translation of policy into practice.

To address these challenges, the study proposes a Provincial Digital Health Governance and Implementation Model that provides an integrated governance framework for strengthening regulatory alignment, institutional coordination, compliance, accountability, and interoperability across provincial health departments. By integrating legislative and regulatory frameworks, health standards, institutional policies, and clearly defined governance responsibilities, the model offers a practical approach to improving the implementation, security, and sustainability of digital health initiatives. The study therefore concludes that strengthening governance capacity, institutional readiness, ICT infrastructure, workforce capabilities, and information security is fundamental to achieving sustainable digital health transformation within South Africa’s public healthcare sector.

Future research should focus on empirically validating the proposed Provincial Digital Health Governance and Implementation Model across different provincial contexts to assess its applicability, adaptability, and effectiveness. Comparative studies across provinces with varying governance structures and resource capacities would provide further insight into implementation challenges and best practices. In addition, future research should examine the influence of interoperability, ICT governance, organizational readiness, and digital skills development on healthcare outcomes and service delivery. Longitudinal studies would provide valuable evidence on the sustainability of digital health governance reforms, while further investigation into the governance implications of emerging technologies, including artificial intelligence and big data analytics, could inform future digital health policy and implementation strategies.

## Data Availability

The data that support the findings of this study are available from the corresponding author upon reasonable request.
